# Effectiveness of brushing teeth in patients with reduced oral hygiene by laypeople: a randomized, controlled study

**DOI:** 10.1186/s12903-021-01590-4

**Published:** 2021-04-30

**Authors:** Anna Greta Barbe, Aya Al-Barwari, Stefanie Hamacher, Renate Deinzer, Ulrike Weik, Michael J. Noack

**Affiliations:** 1 Department of Operative Dentistry and Periodontology, Centre of Dental Medicine, University of Cologne, Kerpener Str. 32, 50931 Cologne, Germany; 2 Faculty of Medicine and University Hospital Cologne, Institute of Medical Statistics and Computational Biology, University of Cologne, 50924 Cologne, Germany; 3 Faculty of Medicine, Institute of Medical Psychology, Justus-Liebig-University Giessen, Klinikstr. 29, 35392 Giessen, Germany

**Keywords:** Brushing support, Care level, Care need, Gerodontology, Nonprofessional, Oral hygiene

## Abstract

**Background:**

To evaluate the success of plaque reduction after external toothbrushing by instructed laypeople versus dental professionals using either a manual or powered toothbrush. Longitudinal, randomized, parallel-group intervention study in periodontitis patients with reduced oral hygiene quality undergoing anti-infective therapy. Patients were randomly and equally assigned to one of four groups: laypeople using a manual or powered toothbrush or dental professionals using a manual or powered toothbrush. Plaque reduction (Quigley–Hein-Index (QHI), Marginal Plaque Index (MPI)), gingivitis (papilla bleeding index), and cleaning time (seconds) were investigated.

**Results:**

Thirty-nine patients participated in the study. Neither the choice of toothbrush (*p* = 0.399) nor the use of a dental professional (*p* = 0.790) had a significant influence on plaque levels achieved. However, multivariate modeling indicated statistically significant differences in the external cleaning time between brushing groups, with longer time required by laypeople (*p* = 0.002) and longer use of the powered toothbrush (*p* = 0.024).

**Conclusion:**

When the ability to carry out personal oral hygiene is reduced, external brushing by dental professionals or instructed laypeople who meet previously defined criteria such as sufficient personal oral hygiene at home could help to fill the emerging dental care gap. A combination of oral hygiene approaches adapted to the individual needs of the patients in need of external help is necessary for optimum oral hygiene.

*Trial registration*: German Clinical Trials register (https://www.germanctr.de; number DRKS00018779; date of registration 04/11/2019).

**Supplementary Information:**

The online version contains supplementary material available at 10.1186/s12903-021-01590-4.

## Introduction

As the average life expectancy increases in Western countries, the number of older people with care needs also increases, including those with oral health problems. In particular, the prevalence of periodontitis and root caries requiring treatment and prosthetic care is high, and new therapeutic concepts are necessary to address these problems [1–5]. The main underlying reason for the evolving need for care is the progressive loss of oral hygiene capability that results from the increasing number of comorbidities and manual restrictions with age, cognitive decline, and often a change from a domestic setting to inpatient care [6]. Many studies have investigated influencing factors on oral hygiene, including the patients themselves, the nursing staff and caregivers [7–9], as well as various concepts and strategies to improve oral hygiene skills and control the increasing burden [10–15]. However, these studies often focus on nursing home residents, whose oral health problems are already in need of treatment. Thus, the resulting oral hygiene regime is problem-oriented, rather than control-oriented, and requires a disproportionate amount of work to improve oral health. In this context, it must be noted that the oral health of older people is not stable but decreases over time. Therefore, it takes more effort to slow the decline in oral health, and even more to improve the situation. We believe that oral intervention should occur when general and oral health first begin to deteriorate, to better control oral health and delay or even prevent the occurrence of more serious dental problems.

Considerations are being given to train laypeople and involve them in oral hygiene at an early stage at an earlier age, before the occurrence of oral health problems that require treatment. They may also support the transition to a phase of life with possible care needs in the outpatient setting. However, there is uncertainty as to whether laypeople could perform regular external toothbrushing and interdental cleaning to the same standard as a dental professional, or even the patient’s own standard, in context of a domestic setting. Clarification of the skills, abilities and services provided by dental professionals in Germany is outlined in Table 1, to better illustrate certain competencies and skills in which nonprofessionals must be trained. In addition to determining who should provide the external toothbrushing, the choice of toothbrush (manual or powered) may also have an effect on plaque reduction. There is broad agreement that powered toothbrushes can be beneficial with older age and limited manual skills [16]. Little is known about whether this is also the case with toothbrushing by third parties.

**Table 1:** Qualification, duration of training, and skills of dental professionals in Germany (ascending qualification grading)

Job title	Qualification and duration of training	Acquired skills
ZFA (Zahnmedizinische/r Fachangestellte/r)	Dual apprenticeship in practice/clinic and vocational college Duration: 3 years	Care of patients Perform prophylaxis measures Assist with treatments Implement hygiene measures Detect infectious diseases Measures to avoid infections Hygiene measures for practice, workplace and own person
ZMP (Zahnmedizinische/r Prophylaxeassistent/in)	Qualification as a ZFA as a prerequisite At least 1 year of professional experience First aid course Expertise in radiation protection Total hours: 736	Anamnesis and diagnosis Instruction and motivation of patients for behavioral education in brushing and flossing Creating an individual oral hygiene plan Implementation of treatment measures within the scope of the included periodontitis therapy (professional tooth cleaning)
ZMF (Zahnmedizinische/r Fachassistent/in)	Qualification as a ZFA as a prerequisite At least 2 years of professional experience First aid course Expertise in radiation protection Total hours: 826	Instruction and motivation of patients for behavioral education Performing professional tooth cleaning Performing prophylactic measures Assistance with treatments
DH (Dentalhygieniker/in)	Qualification as ZMP with at least 400 h and at least 1 year professional experience as ZMP First aid course Completion of three basic course units Expertise in radiation protection Total hours: 800	Anamnesis and diagnosis of patients Diagnosis of oral diseases Implementation of treatment measures within the scope of the included periodontitis therapy (professional tooth cleaning)

To address these questions, our clinical, longitudinal, randomized, parallel-group intervention study in periodontitis patients with reduced oral hygiene quality at home (representing the group of seniors at the beginning of oral hygiene deterioration) undergoing anti-infective therapy aimed to evaluate plaque reduction and time required for external toothbrushing by laypeople or dental professionals using a manual or powered toothbrush. Our hypothesis was that cleaning quality when performed by a third party depends on qualification and competence of the cleaner, as well as the selection of the toothbrushing device.

## Materials and methods

### Ethics

The University of Cologne local ethics review board (19-1407, date 08-19-2019) approved the study, which was registered in the German Clinical Trials register (https://www.germanctr.de; number DRKS00018779; date of registration 04/11/2019). All methods were performed in accordance with the relevant guidelines and regulations (Declaration of Helsinki).

### Study population

The study population comprised patients treated by dental students at the Department of Operative Dentistry and Periodontology, University Hospital Cologne, who had a periodontitis diagnosis, were in need of periodontitis anti-infective therapy, and had reduced domestic oral hygiene. This study population was selected because it appears representative of the group of seniors with an early onset of inadequate oral hygiene. The anti-infective therapy was carried out in accordance with usual medical care and current regulations; initial therapy for periodontitis took place over 3–4 appointments, depending on gingivitis and plaque indices, before the start of closed scaling and root planning therapy. No additional dates were performed for study purposes, but participants were instructed not to brush their teeth 24 h before each study appointment to facilitate evaluation of relevant plaque indices and plaque reduction at the appointments. Participants were told not to eat in the morning before the appointment or drink beverages with sugar or milk (i.e., only black unsweetened tea, black coffee, or water). Patients had to be over 18 years old with at least four remaining teeth, and provided informed written consent. Exclusion criteria were foreseeable loss of residual teeth due to inflammation/loosening diagnosed at the start of the study, toothless jaw, diagnosis of diabetes mellitus with HbA1c > 7.5, endocarditis risk with necessary antibiotic shielding before therapeutic interventions on the teeth, as well as those who were in a dependency/employment relationship with the sponsor or examiner.

### External toothbrushing personnel

In accordance with the core competencies and skills described in Table 1, criteria were determined to reflect a minimum level of competence of brushing and interdental cleaning. Dental personnel trained in cleaning had to have at least level “ZMP” to be included in the dental professional brushing pool. Laypeople had to meet the following requirements after relevant training to be included in the laypeople brushing pool: willingness to take part in brushing training, proof of vocational training, empathy expected in the professional environment, expected empathy in the private environment, personal oral hygiene skills, and the ability to deal professionally with other people. To counteract the bias that the brushing quality with only one person might be person-dependent, four people were included in each pool and one person from each was randomly assigned to a patient according to study appointments and group. Randomization was performed on the basis of pulling sealed envelopes by a person not otherwise involved in the study. The detailed characterization of brushing staff is shown in Additional File 1: Table 2.

#### Training of laypeople

Laypeople were trained to achieve a sufficient brushing competence in September 2019. The training corresponded to the concept of “Oral care in nursing” [17], as recommended by the Federal Dental Council of Germany, where relatives and nursing staff are to be taught how to clean residents. Specific training content included why is it important to brush your own teeth, why you have to be trained to brush teeth, what you should pay attention to when cleaning externally, protective utensils such as gloves, face mask and safety glasses, using the model to show how to brush teeth, correct handling of the toothbrush, holding the cheek with the other hand, cleaning with the brushing technique on inner side, occlusal surfaces and outer surfaces, how to apply dental floss and interdental brushes, and practice brushing on patients and each other, followed by a final examination where clean results by the brushers on their own teeth and in one patient had to be achieved.

### Study design

A full description of study appointments is outlined in Additional File 1: Table 1.

#### Baseline (BL)

On the first study appointment (BL), oral hygiene indices were recorded before patients brushed their own teeth (using their own toothbrush brought from home to the appointment) and immediately afterwards. In accordance with the usual clinical procedures at the University of Cologne, oral hygiene instructions and motivation were provided to the patient, followed by professional tooth cleaning. Subsequently, patients were randomized equally to one of the study groups (laypeople + manual toothbrush, laypeople + powered toothbrush, dental professional + manual toothbrush, dental professional + powered tooth brush). Randomization was performed on the basis of pulling sealed envelopes by a person not otherwise involved in the study.

#### Follow-up 1 (FU-1)

At the second appointment (FU-1), oral hygiene indices were collected initially, followed by external cleaning performed according to the study group. A second measurement of oral hygiene indices was then performed, along with patient motivation and instruction, and a short professional tooth cleaning.

#### Follow-up 2 (FU-2)

On the third appointment, after collection of oral hygiene indices, patients cleaned their own teeth in accordance with BL (i.e., using their toothbrush brought from home) and oral hygiene indices were again measured. This was followed by external cleaning performed according to study group, as per FU-1. To prevent bias, it is important to note that the patient was not cleaned by the same person as at FU-1, but by another person from the corresponding cleaning pool. Oral hygiene indices were again measured, followed by the last short professional tooth cleaning.

### Light-polymerizing plaque test

All oral hygiene indices were determined using a light-polymerizing plaque indicator (Ivoclar Vivodent clinical, Ivoclar Vivadent GmbH, Ellwangen, Germany). The light-polymerizing plaque test (Ivoclar Vivodent clinical, Ivoclar Vivadent GmbH, Ellwangen, Germany), which contains the fluorescent dye fluorescein, was used so that the effect of brushing performance in oral biofilm management was not influenced by the staining of the plaque indicator; stained areas on tooth surfaces can only be recognized by a polymerization lamp. Since the plaque indicator also stains saliva, it is recommended that the patient rinses several times after staining and that tooth surfaces are dried with an air blower. Yellow discoloration is considered plaque under polymerizing light, distinguishing it from the yellow-orange discoloration of tartar.

### Brushing devices

The necessary utensils were prepared prior to the brushing session, and consisted of an powered toothbrush (Oral B Professional Care, Oral-B, Procter & Gamble, Schwalbach, Germany) or a manual toothbrush (Cross Action, Oral-B, Procter & Gamble, Schwalbach, Germany), toothpaste (Oral B Pro-Repair, Oral B, Procter & Gamble, Schwalbach, Germany), interdental brushes in various sizes (“Interdental Brush Original” (TePe Mundhygieneprodukte Vertriebs‐GmbH)), dental floss (Oral-B, Procter & Gamble, Schwalbach, Germany), and SuperFloss (Oral-B, Procter & Gamble, Schwalbach, Germany). The toothpaste was chosen for its 1450 ppm fluoride and mild taste, so we expected sufficient fluoride supply and good acceptance.

### Outcome parameters assessed

#### Clinical characteristics

Age, gender, general illnesses, medication, and allergies were documented at BL.

#### Oral examination

For the oral assessment, the total number of teeth, the decayed, missing, and filled teeth (DMFT) Index, prosthetic situation, periodontal status, and oral hygiene habits were documented at BL, in addition to the relevant hygiene and inflammation indices collected during the course of the study (Table 2).

**Table 2:** Patient and clinical characteristics at baseline

	All patients (N = 39)
Gender	
Female	23(59.0)
Male	16 (41.0)
Year of birth	1964.7 ± 13.2
Age	55.3 ± 13.2
Number of comorbidities	1.4 ± 1.4
Number of prescribed medications	1.9 ± 2.8
Number of teeth	24.3 ± 5.8
Decayed per 28 teeth	4.2 ± 3.6
Missed per 28 teeth	7.7 ± 5.8
Filled per 28 teeth	6.6 ± 4.5
Using a manual toothbrush	23 (76.7)
Using an powered toothbrush	7 (23.3)
Periodontal diagnosis	
Localized	17 (43.6)
Generalized	22 (56.4)
Stage 1	1 (2.6)
Stage 2	13 (33.3)
Stage 3	16 (41.0)
Stage 4	9 (23.1)
Grade A	7 (17.9)
Grade B	17 (43.6)
Grade C	15 (38.5)
Probing depth (mm)	2.7 ± 0.8
Attachment level	4.5 ± 1.6
Papilla bleeding index	0.8 ± 0.8
Quigley–Hein index	1.5 ± 0.5
Marginal plaque index	0.7 ± 0.2

Data are presented as N (%) or mean (± standard deviation), respectively

#### Indices evaluated

The Quigley-Hein index (QHI) and papilla bleeding index (PBI) were obtained as described in detail elsewhere [18–20]. In addition, the marginal plaque index (MPI) was recorded (developed to better differentiate the results of investigated oral hygiene conditions at the gingival margin). Both the vestibular and the oral gingival margin were recorded, with each surface divided into four quadrants of equal size so that two quadrants defined the proximal area of the margin and two the cervical gingival margin tooth surface. The individual quadrants were evaluated numerically using a binary scale, where 0 represents no plaque and 1 represents existing plaque present [21]. To get exact values when collecting the oral health indices (carried out by different people during the study), a calibration was carried out before the study began. The QHI and MPI were measured using the light-polymerizing plaque indicator, and the values were compared; the calibration was successful if the accuracy was 90%. All indices during the course of the study were investigated by the dental students, who were calibrated and the accuracy of 90% assured before the start of the study.

#### Time measurements

The main steps of brushing and interdental cleaning were documented in seconds by a separate dental student who attended the whole session. A a stopwatch program was used (https://www.timeanddate.de/stoppuhr/), where “rounds” could be documented without having a break for resetting the procedure; these time frames were saved and afterwards transferred into the study database. Before starting with the cleaning session, the student was instructed how to use the stopwatch and form and one test run was performed. When planning the study, we decided that differing reaction times of different staff could be neglected, since we did not expect differences of more than two seconds based on experience from prior measurements. The students had one form where measured times were documented, and brushing staff gave verbal feedback when the procedure was finished.

### Sample size

The primary endpoint was the Quigley-Hein-Index, on which the sample size estimate was based [18–20]. To detect an effect size of d = 1 with α = 5% and power = 80% between two unpaired groups and accounting for 15% dropouts resulted in a sample size of n = 40, to be divided equally between the four study groups (laypeople using a manual (n = 10) or powered toothbrush (n = 10) or dental professionals using a manual (n = 10) or powered toothbrush (n = 10)).

### Statistical analysis

Data were analyzed descriptively, and absolute and relative frequencies are presented for qualitative variables, and mean (standard deviation, SD) for quantitative variables. The effect of group, time, and interaction of group and time on PBI (type III sums of squares) was analyzed with a linear mixed model for repeated measurements, with autoregressive first-order heterogenous covariance matrix. Differences in QHI and MPI from pre- to post-brushing were analyzed with a Wilcoxon signed rank test. Group differences in change of QHI and MPI from pre- to post-brushing were compared using the Mann–Whitney U test. Univariate and multivariate logistic regression models were used to assess possible clinically-relevant effects of brusher, brush, brushing time, and baseline score values on QHI and MPI. Individual person-dependent influences were not evaluated. Resulting *p* values are presented for all analyses, all of which are two-sided and considered statistically significant if lower than 5%. Calculations were done with SPSS Statistics 26 (IBM Corp., Armonk, NY, USA). Data were entered twice and reconciled in case of inconsistencies.

## Results

### Patient and clinical characteristics

**Fig. 1:**
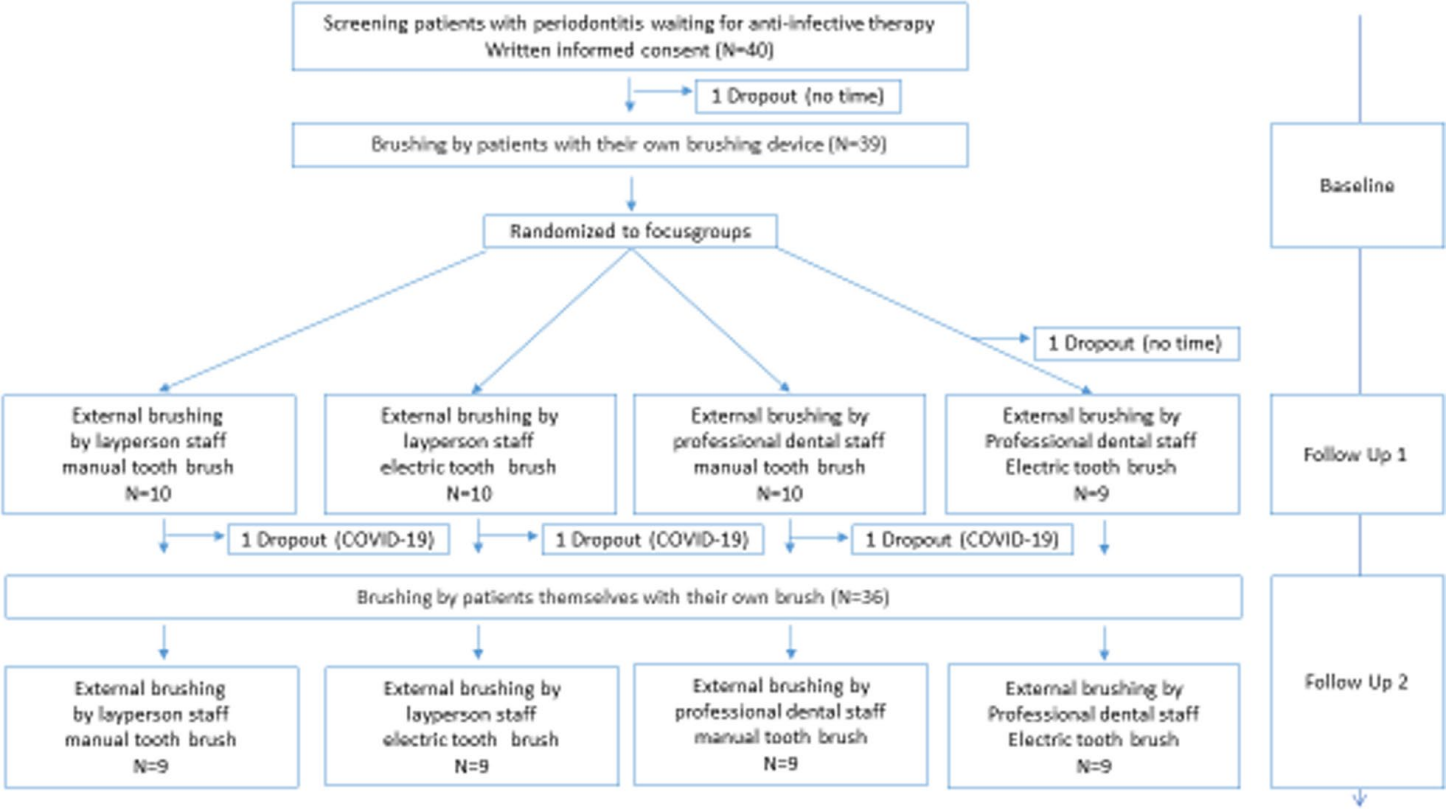
Study flow chart

Overall, 39 patients with periodontitis before anti-infective periodontal therapy participated in the study (Fig. 1). The first patient was included in October 2019 and the last in March 2020. Three patients dropped out before the last appointment due to the COVID19 close-down of the University Hospital of Cologne. Therefore, results were recorded for nine patients in each study group.

Twenty-three (59.0%) patients were women and 16 (41.0%) were men, with a mean age of 56 (SD 13) years (Table 2). Patients suffered from 1.4 (SD 0.37) comorbidities on average, with a mean medication intake of 1.9 (SD 2.8) medications. The mean number of teeth was 24.3 (SD 5.8). According to the new classification of periodontal disease, most patients were classified as stage 2 and 3 and Grade B and C. Regarding oral hygiene habits at home, patients mainly used a manual toothbrush (77%). Regarding their plaque control abilities at baseline, the mean QHI was 1.5 (SD 0.5) and mean MPI was 0.7 (SD 0.2). Periodontal inflammation showed a mean PBI value of 0.8 (SD 0.8) (Table 2).

### Change in periodontal inflammation from baseline to end of study

There was a significant difference in the PBI (*p* < 0.001) over the longitudinal course of the study, but no significant difference between study groups (*p* = 0.691 at FU-1; *p* = 0.423 at FU-2) (Table 3).

**Table 3:** Plaque reduction after self- and/or external brushing by a laypeople or dental professional using a manual or powered toothbrush

	Total N = 39	All	Laypeople/manual toothbrush	Dental professional/manual toothbrush	Laypeople/powered toothbrush	Dental professional/powered toothbrush
PBI-BL	39	0.84 (0.80) 0.56 0.29–1.21	0.65 (0.46) 0.53 0.30–1.03	1.05 (1.21) 0.42 0.17–1.80	0.83 (0.42) 0.53 0.27–1.21	0.81 (0.42) 0.72 0.56–0.89
PBI-FU 1	39	0.48 (0.49) 0.31 0.17–0.57	0.37 (0.29) 0.31 0.1–0.56	0.61 (0.69) 0.32 0.04–0.86	0.41 (0.53) 0.21 0.15–0.44	0.54 (0.38) 0.53 0.29–0.57
*p* value (BL-FU 1)		< 0.001				
PBI-FU 2	39	0.33 (0.33) 0.22 0.09–0.54	0.21 (0.19) 0.20 0.08–0.24	0.41 0.52) 0.20 0.04–0.58	0,28 (0.25) 0.18 0.13–0.38	0,43 (0.26) 0.37 0.19–0.72
*p* value (BL-FU2)		< 0.001				
QHI-BL pre self-brushing	38	1.48 (0.54) 1.46 1.16–1.86	1.55 (0.48) 1.39 1.34–1.77	1.20 (0.64) 1.12 0.77–1.52	1.48 (0.49) 1.59 1.16–1.86	1.66 (0.53) 1.65 1.47–1.92
QHI-BL post self-brushing	38	0.89 (0.52) 0.83 0.57–1.34	0.98 (0.55) 0.90 0.61–1.16	0.60 (0.44) 0.60 0.24–0.61	0.98 (0.55) 1.01 0.54–1.44	1.00 (0.50) 0.85 0.68–1.42
*p* value		< 0.001				
QHI-FU-1 pre external brushing	38	1.14 (0.51) 1.13 0.83–1.46	1.25 (0.70) 1.45 0.52–1.85	1.03 (0.50) 0.92 0.83–1.37	1.17 (0.47) 1.11 0.98–1.21	1.11 (0.37) 1.14 0.82–1.32
QHI-FU-1 post external brushing	38	0.48 (0.32) 0.44 0.210.61	0.60 (0.48) 0.65 0.09–0.89	0.51 (0.29) 0.52 0.38–0.56	0.46 (0.21) 0.48 0.29–0.61	0.32 (0.18) 0.25 0.19–0.41
*p* value		< 0.001				
QHI-FU-2 pre self-brushing	35	1.13 (0.59) 1.02 0.57–1.58	1.0 (0.66) 0.86 0.52–1.48	1.29 (0.74) 1.26 0.55–1.99	1.04 (0.46) 1.07 0.79–1.08	1.19 (0.53) 1.11 1–1.22
QHI-FU-2 Post self-brushing	35	0.51 (0.38) 0.38 0.25–0.68	0.40 (0.29) 0.32 0.3–0.41	0.52 (0.36) 0.52 0.16–0.78	0.56 (0.30) 0.55 0.29–0.71	0.56 (0.54) 0.36 0.25–0.48
*p* value (self-brushing)		< 0.001				
QHI-FU-2 post external brushing	35	0.2 (0.17) 0.15 0.08–0.27	0.20 (0.17) 0.13 0.09–0.27	0.16 (0.11) 0.16 0.06–0.23	0.24 (0.23) 0.16 0.11–0.29	0.21 (0.17) 0.15 0.13–0.19
*p* value (external brushing)		< 0.001				
MPI-BL pre self-brushing	39	0.66 (018) 0.71 0.56–0.79	0.68 (0.17) 0.69 0.59–0.74	0.59 (0.22) 0.62 0.42–0.79	0.65 (0.21) 0.67 0.56–0.84	0.71 (0.12) 0.73 0.71–0.77
MPI-BL post self-brushing	39	0.4 (0.2) 0.4 0.25–0.52	0.42 (0.23) 0.41 0.25–0.44	0.30 (0.18) 0.30 0.17–0.35	0.41 (0.21) 0.38 0.29–0.56	0.48 (0.15) 0.49 0.41–0.56
*p* value		< 0.001				
MPI-FU-1 pre external brushing	39	0.59 (0.22) 0.62 0.47–0.75	0.60 (0.28) 0.70 0.45–0.81	0.62 (0.26) 0.73 0.49–0.76	0.63 (0.18) 0.62 0.53–0.72	0.52 (0.13) 0.56 0.47–0.62
MPI-FU-1post external brushing	39	0.25 (0.16) 0.21 0.14–0.34	0.30 (0.21) 0.28 0.14–0.45	0.28 (0.17) 0.24 0.21–0.35	0.24 (0.11) 0.26 0.14–0.34	0.18 (0.11) 0.15 0.07–0.3
*p* value		< 0.001				
MPI-FU-2 pre self-brushing	36	0.52 (0.18) 0.54 00.37–0.68	0.46 (0.23) 0.42 0.27–0.68	0.56 (0.19) 0.61 0.39–0.71	0.51 (0.15) 0.56 0.41–0.59	0.54 (0.18) 0.52 0.42–0.67
MPI-FU-2 post self-brushing	36	0.27 (0.15) 0.24 0.17–0.36	0.24 (0.18) 0.23 0.1–0.27	0.26 (0.12) 0.29 0.16–0.32	0.30 (0.13) 0.30 0.21–0.4	0.26 (0.17) 0.24 0.17–0.25
*p* value (self-brushing)		< 0.001				
MPI-FU-2 post external brushing	36	0.12 (0.87) 0.09 0.06–0.17	0.11 (0.11) 0.11 0.04–0.12	0.12 (0.08) 0.09 0.05–0.16	0.14 (0.10) 0.11 0.07–0.19	0.10 (0.06) 0.08 0.07–0.12
*p* value (exterbal brushing)		< 0.001				

Data are presented as mean (standard deviation), median (interquartile range). *p* values are from Wilcoxon Signed Ranks tests

*BL* baseline; *FU* follow-up; *MPI* marginal plaque index; *PBI* papilla bleeding index; *QHI* Quigley-Hein index

### Self-brushing ability of patients at baseline

Patients achieved a significant reduction in the mean QHI at BL, from 1.48 (SD 0.54) before self-brushing to 0.89 (SD 0.52) after self-brushing (*p* < 0.001) (Table 3). There was also a corresponding significant reduction in the mean MPI from 0.66 (SD 0.18) to 0.40 (SD 0.20) (*p* < 0.001).

### Effect of external brushing at first follow-up

At the second cleaning appointment (FU-1), where only external brushing took place, a significant reduction in plaque was achieved in all patients. The mean QHI decreased from 1.14 (SD 0.51) before external brushing to 0.48 (SD 0.32) after external brushing (*p* < 0.001). A corresponding significant reduction in the mean MPI was also achieved, from 0.59 (SD 0.22) to 0.25 (SD 0.16) (*p* < 0.001). No difference could be shown in between groups according to laypeople versus dental professional (QHI: *p* = 0.661; MPI: *p* = 0.811) or manual versus poweredtoothbrush (QHI: *p* = 0.179; MPI: *p* = 0.376) (Table 3).

### Effect of adding external brushing to self-brushing regime at second follow-up

On the third cleaning appointment (FU-2), there was a significant reduction in plaque levels after self-brushing took place; mean QHI decreased from 1.13 (SD 0.59) before self-brushing to 0.51 (SD 0.38) after self-brushing (*p* < 0.001), while the MPI decreased from 0.52 (SD 0.18) to 0.27 (SD 0.15) (*p* < 0.001).

**Fig. 2:**
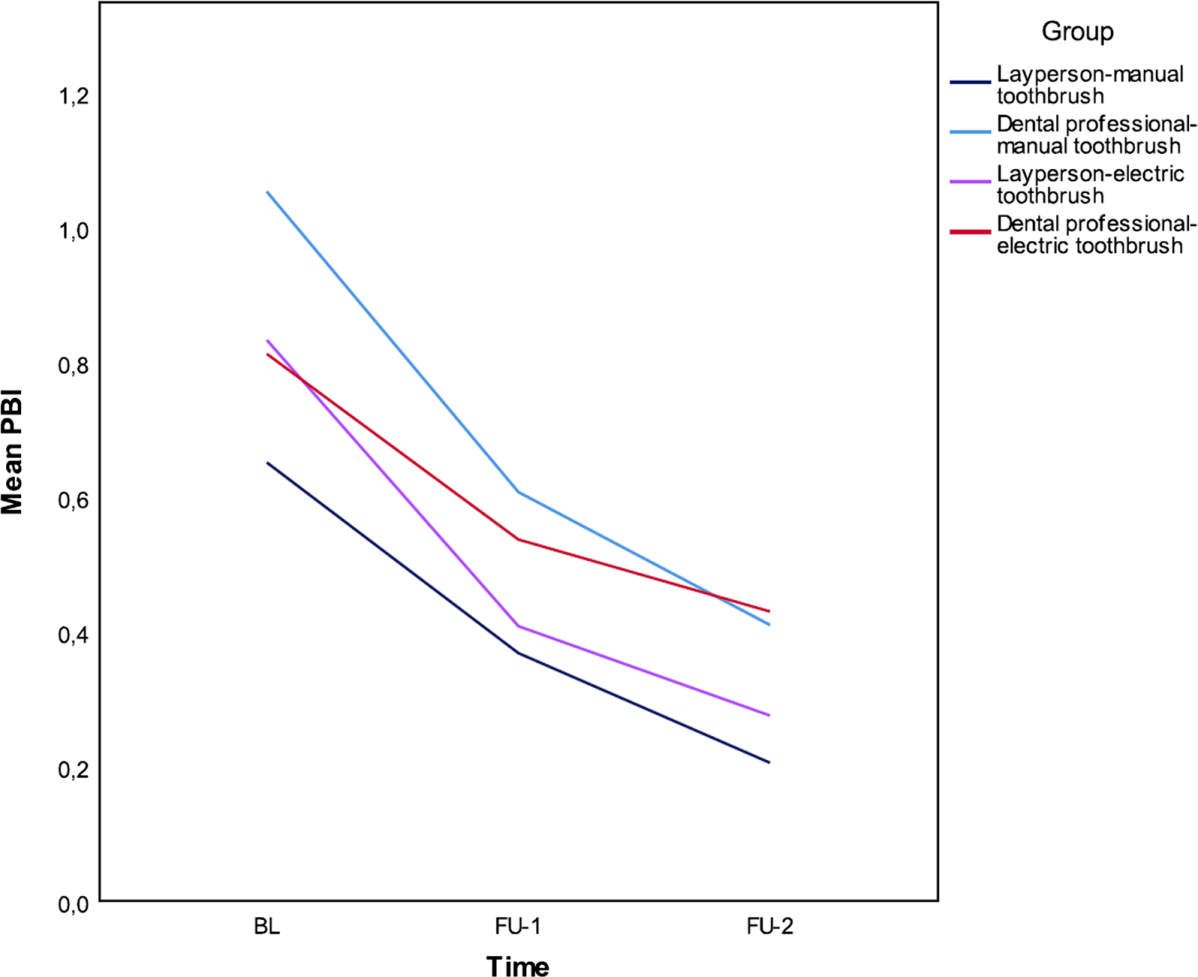
Longitudinal course of gingival inflammation (papilla bleeding index) from baseline to second appointment

External brushing after self-brushing reduced plaque levels even further, with the mean QHI falling to 0.2 (SD 0.17) (*p* < 0.001) and the mean MPI to 0.12 (SD 0.87) (*p* < 0.001) (Fig. 2). No differences could be shown between groups according to laypeople versus dental professional (QHI: self-brushing *p* = 0.106, external brushing *p* = 0.069; MPI: self-brushing *p* = 0.084, external brushing *p* = 0.057) or manual versus powered toothbrush (QHI: self-brushing *p* = 0.792, external brushing *p* = 0.729; MPI: self-brushing *p* = 0.646, external brushing *p* = 1.000) (Table 3).

**Fig. 3:**
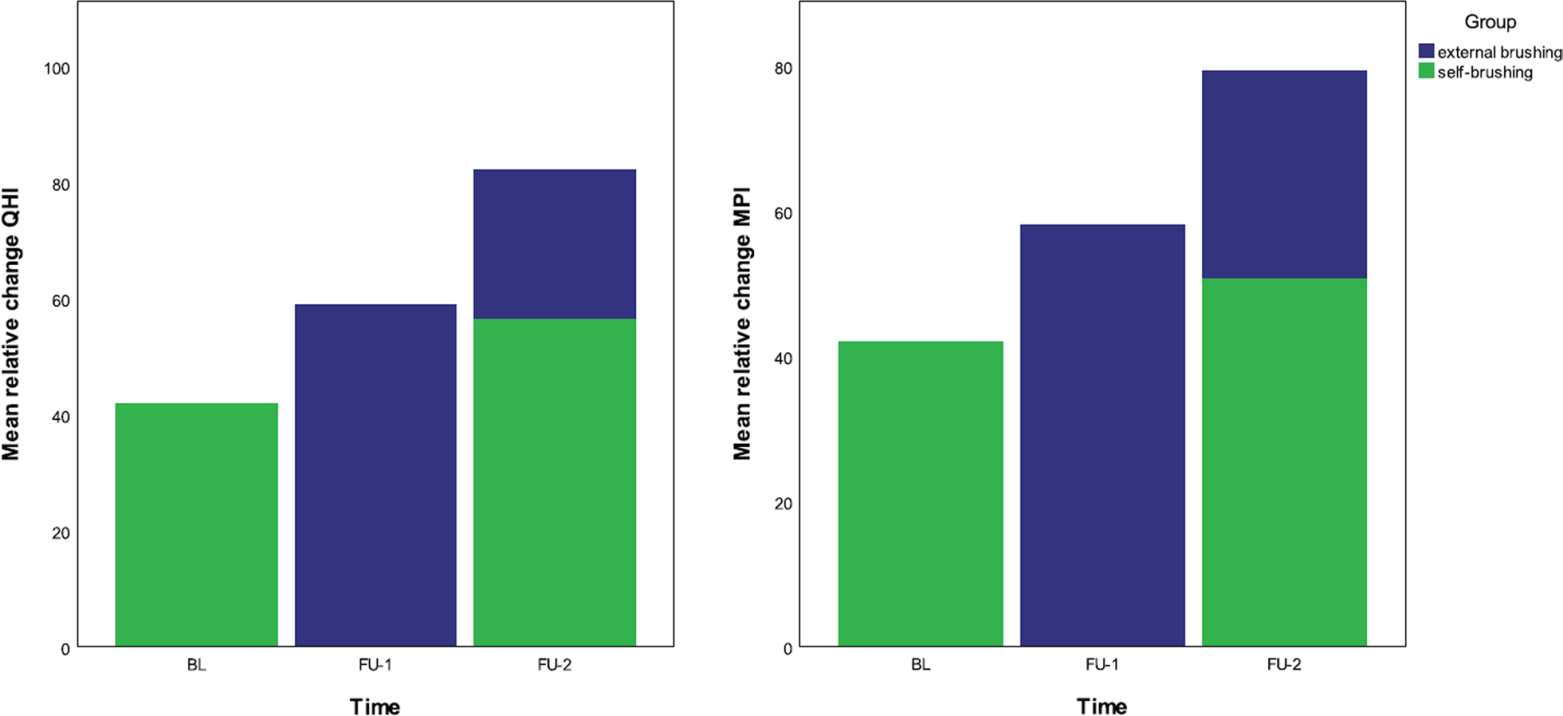
Effect of adding external brushing to self-brushing on plaque reduction measured by Quigley–Hein Index and marginal plaque index. *MPI* marginal plaque index; *QHI* Quigley–Hein index

Overall, self-brushers achieved a 42% ± 23% (QHI) and 42% ± 19% (MPI) reduction in plaque at BL. After external brushing at FU-1, there was a plaque reduction of 59% (SD 17%) (QHI) and 58% (SD 19%) (MPI). At FU-2, self-brushers achieved a greater reduction in plaque than at BL (QHI 56% (SD 17%), MPI 51% (SD 17%). After additional external brushing, this reduction was increased to 82% (SD 11%) (QHI) and 79% (SD 12%) (MPI) (Fig. 3).

### Timeframes for performing brushing and interdental cleaning according to groups

The mean external brushing time across all groups was 250 (SD 123) seconds at FU-1 and 192 (SD 78) seconds at FU-2 (Additional File 1: Table 3). The Kruskal–Wallis test showed that laypeople required significantly more time for external cleaning than dental professionals (*p* = 0.002). In addition, the powered toothbrush was used for a significantly longer time than the manual toothbrush (*p* = 0.024). There was also a significant difference (*p* = 0.002) in the external cleaning time between the four study groups: dental professional/powered toothbrush < dental professional/manual brush < laypeople/manual brush < laypeople/powered brush.

#### Impact of qualification and device

**Fig. 4:**
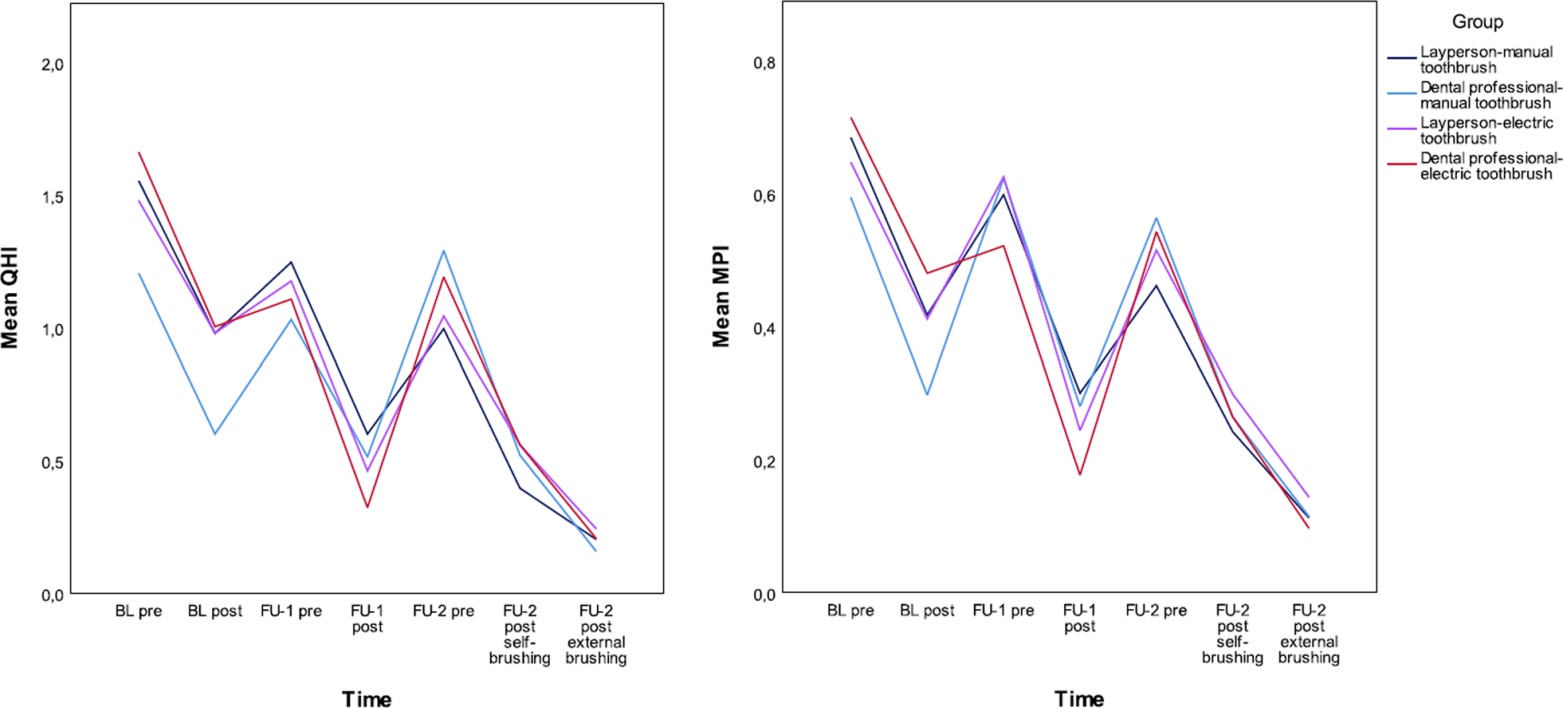
Effect on plaque reduction measured by Quigley–Hein index and marginal plaque index after external brushing by nonprofessionals or dental professionals using a manual or powered toothbrush. *MPI* marginal plaque index; *QHI* Quigley–Hein index

Using the QHI and MPI at FU-1 as dependent variables in the multivariable linear regression model, and the cleaning time, corresponding score at BL, device and qualification as independent variables, there was no significant influence of the device (QHI: *p* = 0.386, MPI *p* = 0.444) or the qualification (QHI: *p* = 0.815, MPI: *p* = 0.740) on the plaque levels achieved (Fig. 4).

## Discussion

The key finding of our study is that after appropriate training, external brushing by laypeople who meet previously defined criteria should reduce plaque to a level that is at least similar to that achieved after self-brushing by the patient and after external brushing by a dental professional among patients with reduced oral hygiene at home. Criteria such as the presence of a professional qualification, professional and personal empathy, the patient’s ability to perform their own oral hygiene, professional appearance, and the willingness to take part in training courses should be considered. Our data also show that the choice of toothbrush used by laypeople and dental professionals does not influence the resulting reduction in plaque. When performing external brushing in our study, laypeople brushed longer than dental professionals, especially when using an powered toothbrush.

There has been much discussion regarding the capabilities of laypeople, methods of recruitment, and the training concepts required to achieve clinical improvements in the general health of patients. Interventions mostly include child care, smoking reduction, or healthy eating approaches in different communities [22–27], and mainly focus on strengthening the capacity of the community to address unresolved health issues [28]. Similarly, the oral health problems in older people with increasingly reduced oral hygiene capability (represented by our study population of periodontitis patients at the beginning of their anti-infectious therapy) also need to be resolved [29–32]. If, as shown in our study, it is possible to train laypeople to ensure a daily and consistently good level of oral hygiene, this early preventive approach could potentially prevent long-term consequences on oral health. However, it is important to understand the factors that may predict the success of such an approach, such as those outlined in a Cochrane review of maternal and health-focused interventions by nonprofessionals [33] where five factors were identified: community relationships, lay health worker program design, intrinsic traits and motivations, work conditions and capacity-building processes, and outcome parameters.

When designing our study, much consideration was given as to how the competence of cleaning staff could be guaranteed. It seemed sensible to define basic skills believed to be necessary to brush the teeth of another person. Many studies have examined the quality of oral hygiene carried out by nursing home staff, as well as how the nursing staff perceived the implementation of oral hygiene [8, 34, 35], and how the patients themselves and their caregivers perceived the cleaning [36]. To address these issues in our study, we defined the core competencies that were necessary in laypeople even before training for the dental competencies took place. It has previously been shown that the knowledge of nursing staff is often insufficient to adequately master their own oral hygiene [37]; thus, a core competence for our study was self-brushing their own teeth at home. In addition, the oral cavity represents an intimate barrier that should not be underestimated; professional and personal empathy is imperative. In our study, this was achieved either through proven professional relations with other people or through nursing experience in the private environment. Whether brushing the teeth of another person should be carried out exclusively by dental specialists should certainly be considered. From a scientific and medical point of view, the general risk of toothbrushing is low and corresponds to correct oral hygiene practices at home, where neither acute nor chronic damage is expected. If a toothbrush, dental floss, or interdental brushes are used improperly as part of dental care, minor gingival injuries, recessions and damage to the hard tooth substance may be possible [38]. The possible spread of intraoral plaque germs in the lungs or bloodstream (bacteremia) has also been described [39], but this risk exists with every chewing process or oral hygiene practices at home. Due to the high prevalence of poor oral hygiene, this risk may slightly be increased in patient populations as in our study, but again corresponds to the risk during oral hygiene practices carried out at home. Deaths associated with external toothbrushing have not been reported in the literature [40]. The well-documented risk of swallowing oral hygiene items to trigger the urge to vomit in eating disorders [41] does not exist with external toothbrushing and has not been described in the literature. Overall, any risk associated with external brushing is much lower than the risk of not providing such assistance.

Regarding the choice of toothbrush and cleaning times, the laypeople in our study cleaned for longer, particularly when using the powered toothbrush. We can only assume that the nonprofessionals wanted to do a particularly good job in the sense of the Hawthorne Effect [42] and so cleaned for a particularly long time. The longer use of the powered toothbrush in laypeople may be related to user-centered preferences. Earlier studies in nursing staff report that it is much easier and more pleasant to brush teeth with powered toothbrush [43]. In this respect, we assume that the brushers had a more comfortable cleaning experience using the powered toothbrush.

In an ideal world, an acceptable standard of oral hygiene among people with reduced oral hygiene abilities would be based on two factors: 1) oral hygiene instruction to address the changing personal oral hygiene capability of the patient, which led to around 60% plaque reduction in our study; 2) additional daily oral hygiene measures provided by other people, such as external cleaning which further reduced plaque by an additional 20% in our study. With an expected decrease in personal oral hygiene ability depending on general health risk factors [44, 45], external toothbrushing could help to fill the emerging dental care gap. Nevertheless, our data demonstrates that even the combination of personal oral hygiene with external toothbrushing does not achieve an oral hygiene standard of 100%. Therefore, further oral hygiene approaches adapted to the individual needs of the patient are necessary. As described in other studies, and critically viewed from a dental perspective [43], external brushing is not about transferring the responsibility for dental tasks to other professional groups. Instead, personal oral hygiene activities that should ideally carried out by the patients themselves, but which may no longer be possible with increasing age, could be performed by nonprofessional staff.

The main limitation of our study is that the periodontitis patients included were most likely to be able to maintain good oral hygiene, but had increased plaque levels due to their domestic cleaning habits and resulting periodontitis. Studies must be carried out using the same methodology to address the target population of people in nursing homes who do not have adequate oral hygiene, based on common diseases and possible cognitive restrictions. Nevertheless, external toothbrushing by laypeople—when combined with a patient’s personal oral hygiene regime, regular dentist visits, and regular professional tooth cleaning sessions—could be one way to increase oral hygiene among populations with reduced oral hygiene capability. Even in patients with reduced oral hygiene capability, the inclusion of laypeople could help to delay oral health problems at the beginning of restrictions in a control-oriented regime, and potentially avoid the need to transition to a control-oriented regime. Another limitation of this study is the fact that oral hygiene instructions and professional cleanings according to the anti-infective therapy have been conducted during the course of the study. However, those cleanings did not influence the before and after examinations before and after the third-party brushing. Another limitation of our study refers to the metric properties of the QHI and the PBI. These are only rank-scaled. Calculation of means and percentages of reduction—though very common in dentistry—are no meaningful mathematical operations within such scales. In our study, we thus employed an additional measure, which is ratio-scaled, i.e. the MPI. This led to comparable results as those found with the QHI. Thus, the degree of distortion due to this violation of mathematical prerequisites appears to be negligible in the present study.

## Supplementary Information

**Additional file 1**.** Supporting Table 1**. Sequence of study dates.** Supporting Table 2**. Characterization according to the skills and competencies regarding the external brushing of the eight brushing people in the study.** Supporting Table 3**. Time (seconds) required for oral brushing and interdental cleaning. Data are presented as mean (standard deviation).

## Data Availability

The datasets used and/or analysed during the current study are available from the corresponding author on reasonable request.
