# Supplementary material for: Effectiveness of brushing teeth in patients with reduced oral hygiene by laypeople: a randomized, controlled study
Source: BMC Oral Health. 2021 Apr 30;21:225. doi: 10.1186/s12903-021-01590-4 (PMC8091671; doi:10.1186/s12903-021-01590-4)
Supplement: Supplementary file 1 — Additional file 1. Supporting Table 1. Sequence of study dates. Supporting Table 2. Characterization according to the skills and competencies regarding the external brushing of the eight brushing people in the study. Supporting Table 3. Time (seconds) required for oral brushing and interdental cleaning. Data are presented as mean (standard deviation). [file 12903_2021_1590_MOESM1_ESM.docx]

**Supporting Table 1.** Sequence of study dates.

| Baseline | Determining oral hygiene indices (PBI, MPI and QHI) (vestibular as well as palatal in each quadrant)  **Patients were told to brush their teeth themselves** with their own cleaning tools, interdental care  Required time is measured  Determining oral hygiene indices (MPI and QHI) (vestibular as well as palatal in each quadrant)  Extraoral and intraoral examination and periodontal status  Participants were motivated and instructed  Professional dental cleaning |
| --- | --- |
| Follow Up 1 | Determining oral hygiene indices (PBI, MPI and QHI) (vestibular as well as palatal in each quadrant)  **External cleaning** with electric or manual brush, interdental care  Required time is measured  Determine oral hygiene indices (MPI and QHI) (vestibular as well as palatal in each quadrant)  Participants were remotivated and reinstructed  Professional dental cleaning |
| Follow Up 2 | Determine oral hygiene indices (PBI, MPI and QHI) (vestibular as well as palatal in each quadrant)  **Participants were told to brush their teeth themselves** with their own cleaning tools, interdental care  Required time is measured  Determine oral hygiene indices (MPI and QHI) (vestibular as well as palatal in each quadrant)  **External cleaning** with electric and manual brush according to FU-1, interdental care  Required time is measured  Determine oral hygiene indices (MPI and QHI) (vestibular as well as palatal in each quadrant)  Participants were remotivated and reinstructed  Professional dental cleaning |

**Supporting Table 2.** Characterization according to the skills and competencies regarding the external brushing of the eight brushing people in the study.

| Gender | Level of education/  qualification | Professional  empathy/ experience | Private  empathy/ experience | Own oral hygiene ability* | Professionalism in dealing with patients |
| --- | --- | --- | --- | --- | --- |
| Dental professionals | | | | | |
| female | Dental hygienist | Patient care | Care for nephews | Given | Given |
| female | ZMP | Patient care | Care of sister with care needs | Given | Given |
| male | Dental student before state exam | Patient care | None | Given | Given |
|  |  |  |  |  |  |
| female | Dental student before state exam | Patient care | None | Given | Given |
| Nonprofessionals | | | | | |
| Female | Medical technical assistant | Yes | Care for father with care needs | Given | Given |
| Female | Foreign language correspondent  Secretary university hospital | Yes | Own child | Given | Given |
| Female | Midwife | Yes | Own children, care for neighbour | Given | Given |
| Male | Physiotherapist | Yes | None | Given | Given |

*****Own oral hygiene skills demonstrated by brushing as part of the training, based on QHI values collected before and after self-brushing (score must be <1 to participate in the study) and, if necessary, optimization of brushing deficits. QHI, Quigley-Hein index.

**Supporting Table 3:** Time (seconds) required for oral brushing and interdental cleaning. Data are presented as mean (standard deviation).

|  | Total  N=39 | All study patients | | Non-professional/ manual  toothbrush | | Dental professional/ manual toothbrush | | Non-professional/ electric toothbrush | | Dental professional/ electric toothbrush | |
| --- | --- | --- | --- | --- | --- | --- | --- | --- | --- | --- | --- |
| BL brush | 39 | 143 | (42) | 147 | (34) | 145 | (54) | 152 | (48) | 126 | (24) |
| BL IDC | 14 | 80 | (34) | 95 | (35) | 63 | (5) | 75 | (52) | 94 | (32) |
| FU-1 brush | 39 | 250 | (123) | 258 | (68) | 158 | (40) | 374 | (163) | 206 | (49) |
| FU-1 IDC | 38 | 121 | (59) | 146 | (53) | 72 | (29) | 161 | (59) | 104 | (47) |
| FU-2  self-brush | 36 | 149 | (51) | 146 | (41) | 167 | (48) | 131 | (35) | 151 | (74) |
| FU-2  self IDC | 17 | 81 | (52) | 89 | (32) | 66 | (28) | 79 | (44) | 86 | (88) |
| FU-2  external brush | 36 | 192 | (78) | 179 | (81) | 165 | (50) | 259 | (101) | 165 | (26) |
| FU-2  external IDC | 36 | 126 | (64) | 145 | (44) | 100 | (56) | 94 | (45) | 165 | (82) |

BL, baseline; FU, follow-up; IDC, Inter-dental cleaning.
